# 

**Published:** 1860-10-01

**Authors:** 


### CONTENTS.

| ART. | | PAGE |
|---|---|---|
| | Psychological Quarterly Retrospect | lxiii.—lxxvi. |
| I. | The Parliamentary Inquiry Concerning Lunatics | 437 |
| II. | On the Amelioration of Races by Education and Intermarriage | 458 |
| III. | The Status of Crime in 1859 | 484 |
| IV. | The Modern Drama: a Contribution to Mental Dietetics | 502 |
| V. | Braïdism | 516 |
| VI. | The State of Lunacy in England | 525 |
| VII. | Modern Developments of the Marvellous | 534 |
| VIII. | Statistics of Insanity in the United States | 554 |
| IX. | Recent Legislation on Criminal Lunatics | 586 |
| | Foreign Psychological Literature | 588 |
| | Gheel.—Letter from Dr. Willers Jessen | 612 |
| | Prize Essay on Cretinism | 613 |

INDEX TO VOLUME XIII.

